# Autism in adult psychiatric out-patients: self-reported suicidal ideation, suicide attempts and non-suicidal self-injury

**DOI:** 10.1192/bjo.2023.553

**Published:** 2023-09-07

**Authors:** Johan Nyrenius, Margda Waern, Jonas Eberhard, Mohammad Ghaziuddin, Christopher Gillberg, Eva Billstedt

**Affiliations:** Gillberg Neuropsychiatry Centre, Institute of Neuroscience and Physiology, Sahlgrenska Academy, University of Gothenburg, Sweden; Adult Psychiatric Clinic of Helsingborg, Region Skåne, Sweden; and Department of Clinical Sciences Lund/Helsingborg, Lund University, Sweden; Sahlgrenska Suicide Studies, Institute of Neuroscience and Physiology, Sahlgrenska Academy, University of Gothenburg, Sweden; Adult Psychiatric Clinic of Helsingborg, Region Skåne, Sweden; and Department of Clinical Sciences Lund/Helsingborg, Lund University, Sweden; Department of Psychiatry, University of Michigan, Michigan, USA; Gillberg Neuropsychiatry Centre, Institute of Neuroscience and Physiology, Sahlgrenska Academy, University of Gothenburg, Sweden; and Child Neuropsychiatric Clinic, Sahlgrenska University Hospital, Sweden

**Keywords:** Autism, suicide, non-suicidal self-injury, adults, out-patient psychiatry

## Abstract

**Background:**

The prevalence of self-reported suicidal ideation, suicide attempts and non-suicidal self-injury (NSSI) remains unclear among adults with autism unrecognised in childhood who attend psychiatric services.

**Aims:**

We aimed to estimate the prevalence of suicidal ideation, suicide attempts and NSSI; identify factors associated with suicide attempts and NSSI; and describe NSSI in this group.

**Method:**

Sixty-three new patients at an adult psychiatric out-patient clinic (57% women, mean age 32 years) who met full (*n* = 52) or subthreshold (two A criteria and minimum of two B criteria; *n* = 11) DSM-5 criteria for autism spectrum disorder were included in the study. Clinical assessments included overall diagnostic review, Paykel's questions on passive and active suicidality, evaluation of NSSI with the Functional Assessment of Self-Mutilation, and results of cognitive tests. One follow-up of medical records was made.

**Results:**

In this sample of psychiatric out-patients identified as first having autism in adulthood, almost a third (31%) of patients reported suicidal ideation during the past month, 86% had lifetime suicidal ideation and 25% reported at least one suicide attempt. Factors associated with suicide attempts included hazardous or harmful alcohol use and/or drug-related problems, and severity of depression. A total of 44% reported NSSI. Factors associated with NSSI were female sex, history of suicidal plans and antisocial personality disorder. Substance or alcohol use were often overlooked, especially in women.

**Conclusions:**

Suicidal ideation, suicide attempts and NSSI were very common in adults with autism who were recently referred to an out-patient psychiatric service. Suicidal ideation and NSSI were more common than suicide attempts. Clinicians should always consider suicidal ideation and NSSI in adult psychiatric patients with autism.

Suicide – ‘death caused by injuring oneself with the intent to die’^1^ – occurs across different cultures and age categories. Around 800 000 people worldwide die from suicide every year,^2^ constituting 1.3% of deaths globally.^3^ Psychiatric disorder is a major risk factor for suicide, and the prevention of suicide is an especially relevant issue in psychiatric settings.^4^

Although often identified during childhood, autism or autism spectrum disorder (ASD) has gained increasing attention in adult psychiatric settings. Today, autism is widely considered to be a lifelong condition, and an increasing number of adults in contact with out-patient psychiatric services are diagnosed with autism in adulthood. The prevalence of autism in the general population is between 1 and 1.5%.^5^ Psychiatric disorders are highly prevalent in individuals with autism,^6–8^ and autism prevalence estimates in psychiatric populations published during the past 10 years range between 2.5 and 20%.^9–12^ Although many autistic people prefer identity-first language (i.e. ‘autistic person' rather than ‘person with autism'), many do not. We choose not to use identity-first language, since ASD is a diagnosable condition according to DSM-5/ICD-11.

Suicide and suicidality are more common in psychiatric populations than in the general population. Common risk factors for suicide among psychiatric patients are prior history of suicide attempts, feelings of hopelessness, impulsivity and aggression, adverse childhood experiences (ACEs), severe psychopathology and physical/somatic disorders.^4^ Higher rates of psychiatric comorbidity are also associated with higher suicide risk.^4^

## Suicidality and non-suicidal self-injury in adults with autism

Increased risk of suicide attempts and death by suicide has been reported in adults with autism when compared with adults without autism in register studies.^13,14^ A recent study reported elevated rates of suicidal ideation in young adults (18–25 years of age) with autism presenting at emergency departments compared with young adults without autism, based on register data.^15^ Another recent study, using data from medical charts, reported suicidal thoughts and behaviours to be common among adults with autism presenting at psychiatric emergency departments, although suicide-related issues were not always the reason for the presentation.^16^ However, studies based on patient interviews focusing on the prevalence of self-reported suicide ideation and risk factors for suicide attempts in adults with autism without intellectual disability in wider age groups are, to our knowledge, limited to one British study on adults with Asperger's syndrome,^17^ set in a specialist clinic, so results cannot be extrapolated to general psychiatric services. Commonly known risk factors for suicide and suicidality in the general population, routinely addressed in suicide risk assessments, include history of suicidal behaviour and mental illness; family history; impulsivity; alcohol or substance misuse; serious physical/somatic illness; legal, vocational and financial problems; history of ACEs; being a victim and/or perpetrator of violence and social isolation.^1^ Risk factors for suicide and suicidality in adults with autism seem similar to risk factors in adults without autism, although a number of factors have been suggested as especially important and/or specific in adults with autism. The general traits of autism – that is, deficits in communication and reciprocal social interaction, and restricted and/or repetitive behaviours – as measured by the Autism Spectrum Quotient, Short Form (AQ-S), have been proposed to exacerbate the social risk factors.^18^ Autism has, in itself, been proposed as a risk factor for suicidal behaviour, mediated by depressive symptoms, quality and frequency of social interaction, and educational attainment.^19^ Depressive rumination and low self-esteem,^20^ social isolation,^18^ depression and alexithymia,^21^ degree of co-occurring psychiatric conditions and female gender^13^ have been reported as risk factors for suicidal behaviours in adults with autism. Unmet support needs, camouflaging and non-suicidal self-injury (NSSI) have been reported to predict higher results on self-rating scales for suicidality in adults with autism.^22^ NSSI has been reported as predictive of both the occurrence of suicide attempts and of more numerous suicide attempts in adults with autism, who, compared to adults without autism, have also been suggested to report a reduced fear of death and more mental rehearsal of suicide, which in turn was a mediating factor for lifetime suicide attempts.^23^

NSSI has been defined as ‘the direct, deliberate destruction of one's own body tissue in the absence of suicidal intent’.^24^ An important difference between NSSI and other forms of self-injurious behaviours is that people who engage in NSSI do not intend to end their own life, and NSSI is often performed without present suicidal ideation.^25^ Different diagnostic categories show markedly differing prevalence rates of NSSI, with the highest prevalence often reported among persons diagnosed with borderline personality disorder (lifetime prevalence exceeding 63%)^26^ and eating disorders (lifetime prevalence 27%).^27^ The most commonly stated reason for engaging in NSSI is to regulate emotions.^28^ NSSI among adults with autism without intellectual disability has only been reported in a small number of studies. Three (online) surveys^22,23,29^ have suggested lifetime prevalence rates of NSSI at 50–74% in adults with autism; two of the surveys^22,23^ found lifetime prevalence rates of NSSI to be higher in adults with autism than in adults without autism. The functions of, or reasons for engaging in, NSSI in adults with autism have so far been reported to be emotional regulation,^30–32^ but also in some cases sensory stimulation.^32^ In adults with or without autism, NSSI and suicidal behaviour are associated.^31,33^

Increasing evidence speaks for a high rate of ASD in adult psychiatric out-patient populations.^9,10^ The high rates of ASD in adult psychiatric out-patient populations might possibly be an effect of psychiatric disorders being more prevalent in adult autism populations than in the general population.^9^ ‘Camouflaging’ of autistic traits has also been suggested as a possible risk factor for psychiatric disorders, especially in women.^34^ More and more studies are published regarding NSSI and suicidality in adults with autism; however, to the best of our knowledge, no studies have so far reported on suicidality or NSSI in adult psychiatric out-patients with autism. The aims of the present study were (a) to estimate the prevalence of self-reported suicidal ideation, suicide attempts and NSSI; (b) to identify factors associated with suicide attempts and NSSI; and (c) to characterise NSSI behaviours in adult psychiatric out-patients with autism.

## Method

### Procedure

This study is part of a larger cross-sectional research project on adult (age ≥18 years) psychiatric patients with autism in psychiatric out-patient care, which collected data on newly referred patients at an adult psychiatric out-patient clinic in Helsingborg, Sweden (catchment area population in eligible age range: 215 000), between 1 January 2019 and 31 December 2020. Newly referred patients were invited to complete a screening questionnaire for autism. Patients who screened positive for autism and a screen-negative comparison group were invited to participate in an in-depth clinical assessment lasting 2 h, using validated instruments. Follow-ups of the participants’ medical records regarding clinical diagnoses and occurrence of any deaths were performed in late August and early September 2021. The follow-ups were between 7 and 17 months after study participation. Screening was performed at the psychiatric assessment unit, where new catchment area patients (without psychiatric contact during the past 6 months) are assessed, and at the substance use disorders unit. Referrals with psychotic illness or age ≥67 years were not included as these persons are treated at other clinics. For comprehensive information about the screening and attrition process, please see previously published studies from the project.^9,35^

### Ethics approval and participant consent

The authors assert that all procedures contributing to this work comply with the ethical standards of the relevant national and institutional committees on human experimentation and with the Helsinki Declaration of 1975, as revised in 2008. All procedures involving human patients were approved by the Regional Ethics Review Board in Lund, Sweden (reference number 2018/740). All participants provided written informed consent.

### Participants

Seventy-nine patients who screened positive and nine patients who screened negative from the psychiatric assessment unit, and two patients who screened positive from the substance use disorders unit, took part in in-depth assessments. Out of these, 63 patients (hereafter referred to as ‘participants’) were assigned a research diagnosis of ASD (according to the DSM-5 criteria;^36^ *n* = 52) or subthreshold ASD (defined as meeting two rather than all three A criteria and at least two B criteria for ASD, according to the DSM-5; *n* = 11). The 27 participants who did not meet criteria for ASD or subthreshold ASD were not included in the present report, given the limited size of the group, making it unsuitable for use as control group. The final cohort for this study comprised these 63 participants (27 men and 36 women) meeting criteria for ASD or subthreshold ASD, ranging from 18 to 65 years of age, with a mean estimated intelligence quotient (IQ) ranging from 80 to 130. Of the 63 participants, 26 were referred for assessment of neurodevelopmental disorders (of whom ten had autism specifically mentioned in the referral), 15 were referred for depression or other mood disorders, eight were referred for anxiety disorders, seven were referred for other psychiatric disorders (severe or post-traumatic stress, personality disorder, suspected psychotic disorder or substance use disorder) and the final seven did not have a specified reason for referral (e.g. referred for a thorough psychiatric assessment). Further descriptive characteristics of the cohort are shown in Table 1. Two of the participants had a prior diagnosis of ASD before participating in the study. For comprehensive information about diagnostic procedures for ASD and other neurodevelopmental and psychiatric disorders and diagnoses, see ‘In-depth assessment instruments’ below and previously published studies from the project.^9,35^

**Table 1:** Characteristics of the study sample

Characteristic	Men (*n* = 27)	Women (*n* = 36)	Total (*N* = 63)
Age (years), mean (s.d.)	35.8 (12.1)	29.1 (8.2)	32.0 (10.5)
GAF, mean (s.d.)	49.3 (7.1)	49.7 (5.9)	49.5 (6.4)
History of depression, *n* (%)	25 (93%)	34 (94%)	59 (94%)
Persistent depressive disorder,^a^ *n* (%)	10 (37%)	4 (11%)	14 (22%)
Antisocial personality disorder,^b^ *n* (%)	2 (7%)	5 (14%)	7 (11%)
Number of DSM-5 non-mood diagnoses,^b^ excluding ASD, mean (s.d.)	3.7 (1.6)	4.3 (1.8)	4.1 (1.7)
ASDI result, mean (s.d.)	23.4 (6.2)	23.5 (5.9)	23.5 (6.0)
IQ estimate, mean (s.d.)	97.0 (9.1)	99.4 (12.3)	98.4 (11.0)
PSI, mean (s.d.)	93.4 (11.6)	100.7 (14.9)	98.4 (13.7)
Delayed/deviant language development, *n* (%)	9 (33%)	14 (39%)	23 (36%)
Motor difficulties in childhood, *n* (%)	11 (41%)	21 (58%)	32 (51%)
Tic disorder, *n* (%)	14 (52%)	23 (64%)	37 (59%)
Lifelong sensory hyper- or hypo-reactivity, *n* (%)	20 (74%)	34 (94%)	54 (86%)
Hazardous or harmful alcohol use and/or drug-related problems	6 (22%)	13 (36%)	19 (30%)
Clinical diagnosis of alcohol/drug dependence or harmful use (ICD-10)^c^ at follow-up	6 (22%)	2 (6%)	8 (13%)

Data presented as mean (s.d.) or *n* (%). GAF, Global Assessment of Functioning; ASD, autism spectrum disorder; ASDI, Asperger Syndrome (and High-Functioning Autism) Diagnostic Interview; PSI, Processing Speed Index.

a. As defined in the DSM-5.

b. According to the Mini-International Neuropsychiatric Interview.

c. Any diagnosis of dependence and/or harmful use in the F10 criteria range, regardless of substance. No differences between male and female participants were found in any variable (*t*-test, Mann–Whitney *U*-test, chi-squared test and Fisher's exact test; Bonferroni-corrected *α* = 0.003).

### Instruments

#### Screening instruments

Initially, the Ritvo Autism Asperger Diagnostic Scale – Revised (RAADS-R^37^) was used, but a high attrition rate meant that this was replaced by the shorter RAADS-14 Screen^38^ on 1 November 2019. Of the 63 participants, 27 were screened with the RAADS-R and 36 were screened with the RAADS-14. RAADS-R scores (range 0–240) of at least 50 and RAADS-14 scores (range 0–42) of at least 14 were considered positive for autism. The screening instruments were not used to assign diagnoses of ASD.

#### In-depth assessment instruments

The Mini-International Neuropsychiatric Interview (M.I.N.I) version 7.0.1, including the attention-deficit hyperactivity disorder supplement,^39^ is a short and structured diagnostic interview designed to identify occurrence of the most common psychiatric disorders (including antisocial personality disorder), according to DSM-5 criteria. The sections on substance/alcohol misuse were replaced by the Alcohol Use Disorders Identification Test (AUDIT^40^) and the Drug Use Disorders Identification Test (DUDIT^41^). Cut-off scores for the AUDIT (‘hazardous or harmful alcohol use’) are 8 points for men and 6 points for women.^42^ Cut-off scores for the DUDIT (‘drug-related problems’) are 6 points for men and 2 points for women.^41^

The five Paykel questions^43^ were used to assess active and passive suicidal ideation. These include (a) ‘Have you ever felt that life was not worth living?’; (b) ‘Have you ever wished that you were dead? For instance, that you would go to sleep and not wake up?’; (c) ‘Have you ever thought of taking your own life, even if you would not really do it?’; (d) ‘Have you ever reached the point where you seriously considered taking your own life, or perhaps made plans how you would go about doing it?’ and (e) ‘Have you ever made an attempt to take your life?’. Participants with affirmative responses were asked to report the most recent occurrence (‘more than one year ago’, ‘past year’, ‘past month’ and ‘past week’). Participants with a history of suicide attempt(s) were asked to report the number of suicide attempts and methods employed. History of NSSI was assessed with one screening question (‘Have you ever done something to hurt yourself, or done something dangerous where you could have died, but without ANY intention to take your life?’), answered with ‘yes’ or ‘no’. The Paykel questions and the NSSI screening question were presented as a written questionnaire, with verbal instructions that the participant was free to ask the researcher (who was present for the entire time) for clarification if needed. Participants with an affirmative response to the NSSI screening question were asked to complete the Functional Assessment of Self-Mutilation (FASM^44^), which includes 11 self-injury behaviours, and when NSSI is present, a further 22 items about the reason for self-injury.

ASD features/traits were assessed with the Asperger Syndrome (and high-functioning autism) Diagnostic Interview (ASDI^45^), a semi-structured interview consisting of 20 questions, each rated 0–2. Total scores may range from 0 to 40. The ASDI is designed to be used by a clinician as an aid in preliminary diagnostic decisions. Research diagnoses of ASD were not assigned based on the ASDI score; the ASDI was rather employed as a structured means of assessing the core features/traits of ASD (please see below for description of the diagnostic procedure for ASD). The ASDI has shown good inter- and intrarater reliability, and acceptable validity.^45^ Functional level was rated with the Global Assessment of Functioning (GAF^46^), which is a crude and widely used assessment of psychosocial functioning level ranging from 0 to 100, with higher scores indicating better psychosocial functioning. The GAF rating was made by the interviewer based on all available information. Tic disorder was diagnosed in participants showing clear tics during the interview and/or self-reporting tics according to DSM-5 criteria.^36^ Sensory hyper- or hypo-reactivity was assessed clinically on the basis of participants’ self-report. To classify ‘sensory hyper- or hypo-reactivity’, problems had to have been present since childhood.

Sociodemographic and clinical characteristics were assessed with a self-report questionnaire that covered living conditions, relationships, educational background, vocational status, economic situation, contacts with healthcare and social services, prior neurodevelopmental or psychiatric assessments, and medication. Cognitive parameters were assessed with three subtests (Matrix Reasoning, Coding and Symbol Search) from the Wechsler Adult Intelligence Test, Fourth Edition (WAIS-IV^47^). The subtests Matrix Reasoning and Coding have been shown to accurately estimate IQ level.^48^ Results from the subtests Coding and Symbol Search were used to calculate the Processing Speed Index score.

Details about psychiatric disorders, alcohol/drug consumption, sociodemographic characteristics, functional level, screening results and ASD features/traits have been presented elsewhere.^9,35^ All available data from the in-depth assessments, including the clinical assessments, were used to assign a research diagnosis of ASD according to DSM-5 criteria. The diagnoses were assigned by clinicians with extensive experience diagnosing ASD and psychiatric disorders. Conjoint validation of the ASD diagnoses was performed by four of the authors (J.N., M.G., C.G. and E.B.), using a subsample of five participants. More details regarding ASD assessment have been published previously.^9^

### Statistical analyses

All statistical analyses were performed with IBM SPSS for Windows, version 25. The statistical significance criterion was set *a priori* to (alpha) *P* = 0.05. Between-group comparisons were performed with Welch's *t*-test (see below) or Mann–Whitney *U*-test, depending on whether the variables tested were normally distributed or not. For categorical data, chi-squared or Fisher's exact test (for 2 × 2 tables) were used. Logistic regression analyses were performed to estimate relationships between the outcome variables ‘suicide attempt’ and ‘NSSI’ and their different predictors, and to further analyse relationships between significant predictors.

Known risk factors for suicide attempts in adults with or without ASD (age, sex, having had economic support from social services, experience of psychologically traumatic events (as defined in the M.I.N.I)), number of non-mood psychiatric diagnoses (degree of psychiatric comorbidity), hazardous or harmful alcohol use and/or drug-related problems, number of depression symptoms during the most severe depressive episode, ASDI score (as a measure of degree of ASD features/traits) and cognitive factors (IQ estimate and Processing Speed Index) were analysed by forward stepwise logistic regression, as potentially associated factors for suicide attempts among adult psychiatric out-patients with autism.

Previously reported risk factors for NSSI, such as age and female sex;^49^ history of active suicidal ideation and suicidal behaviour;^49,50^ and antisocial personality disorder^50^ were analysed by forward stepwise logistic regression as potential factors associated with NSSI. We added ASDI score (as a measure of degree of ASD features/traits) and occurrence of attention-deficit hyperactivity disorder (ADHD).

ASDI outcomes (as a measure of degree of ASD features/traits) were compared across participants with and without ongoing depression at the time of the in-depth assessment, to check if diagnoses of ASD could have been influenced by presence of symptoms of depression. The comparison was performed with Welch's *t*-test because the variance of the ASDI results were not similar enough in the two groups, according to Levene's test (*P* < 0.05).

## Results

### Prevalence of suicidal ideation and suicide attempts

Out of all participants, 94% had experienced feelings of life not being worth living, 79% had experienced wishing to be dead, 86% had experienced thoughts of taking their own lives, 63% had seriously considered taking their own lives and 25% had experience of at least one suicide attempt. Further details regarding suicidal thoughts and behaviours are presented in Table 2. Active suicidal ideation (‘Have you ever thought of taking your own life?’) was more common in the longer perspective than passive suicidal ideation (‘Have you ever wished that you were dead?’). During the past year, 58% of the participants had had thoughts of death or of taking their lives, whereas 29% had seriously considered and 7% had attempted suicide. A fourth of the participants had a history of at least one suicide attempt. Two of the participants presented with high risk of suicide at the time of the interview and were put in contact with psychiatric emergency services. In the subgroup with a history of suicide attempt(s), 19% met the DSM-5 criteria for persistent depressive disorder and all had a history of depression (any type). Poisoning was the most common method, noted in more than half (ten out of 16) of the suicide attempts. In three of these cases, an additional method (attempted drowning, cutting, jumping in front of a train) was used. The remaining six attempts employed cutting (*n* = 3), hanging (*n* = 2) or strangulation (*n* = 1). No deaths by suicide were recorded in medical records during follow-up.

**Table 2:** Prevalence of suicidal feelings in adult psychiatric out-patients with autism, stratified by time

	Total	Men	Women
	*(N* = 63) *n* (%)	(*n* = 27) *n* (%)	(*n* = 36) *n* (%)
‘Have you ever felt that life was not worth living?’			
Past week	14 (22%)	7 (26%)	7 (19%)
Past month	11 (17%)	6 (22%)	5 (14%)
Past year	15 (24%)	6 (22%)	9 (25%)
More than a year ago	19 (30%)	6 (22%)	13 (36%)
Lifetime	59 (94%)	25 (93%)	34 (94%)
‘Have you ever wished that you were dead? For instance, that you would go to sleep and not wake up?’			
Past week	10 (16%)	6 (22%)	4 (11%)
Past month	10 (16%)	3 (11%)	7 (19%)
Past year	13 (21%)	8 (30%)	5 (14%)
More than a year ago	17 (27%)	3 (11%)	14 (39%)
Lifetime	50 (79%)	20 (74%)	30 (83%)
‘Have you ever thought of taking your own life, even if you would not really do it?’			
Past week	9 (14%)	5 (19%)	4 (11%)
Past month	11 (17%)	5 (19%)	6 (17%)
Past year	17 (27%)	11 (41%)	6 (17%)
More than a year ago	17 (27%)	3 (11%)	14 (39%)
Lifetime	54 (86%)	24 (89%)	30 (83%)
‘Have you ever reached the point where you seriously considered taking your own life, of perhaps made plans how you would go about doing it?’			
Past week	1 (2%)	1 (4%)	0 (0%)
Past month	6 (10%)	1 (4%)	5 (14%)
Past year	11 (17%)	7 (26%)	4 (11%)
More than a year ago	22 (35%)	8 (30%)	14 (39%)
Lifetime	40 (63%)	17 (63%)	23 (64%)
‘Have you ever made an attempt to take your life?’			
Past week	0 (0%)	0 (0%)	0 (0%)
Past month	1 (2%)	0 (0%)	1 (3%)
Past year	3 (5%)	1 (4%)	2 (6%)
More than a year ago	12 (19%)	4 (15%)	8 (22%)
Lifetime	16 (25%)	5 (19%)	11 (31%)

### Factors associated with suicide attempts

Forward stepwise logistic regression was performed with suicide attempt as outcome variable. The resulting model included three of the variables: hazardous or harmful alcohol use and/or drug-related problems, traumatic experiences (experience of psychologically traumatic events as defined in the M.I.N.I) and number of depressive symptoms during most severe depressive episode. A follow-up logistic regression analysis was made, showing that traumatic experiences were associated with hazardous or harmful alcohol use and/or drug-related problems (odds ratio 5.9, *P* = 0.03). Therefore, the trauma variable was removed from the regression model. The final model consisted of hazardous or harmful alcohol use and/or drug-related problems (AUDIT or DUDIT score above cut-off; odds ratio 6.0, *P* < 0.01), and number of depression symptoms during most severe depressive episode (odds ratio 2.2, *P* < 0.05). The model explained between 21.3 and 31.1% of the variance in occurrence of suicide attempts (Cox and Snell *R^2^* = 0.213; Nagelkerke *R^2^* = 0.311). Details from the regression are available in the supplementary material.

### NSSI behaviours

**Fig. 1:**
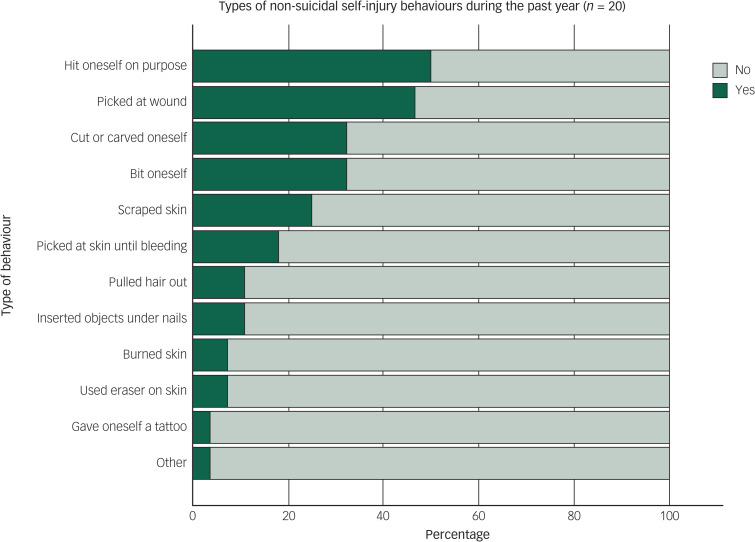
Prevalence of different types of non-suicidal self-injury behaviours during the past year, according to the Functional Assessment of Self-Mutilation (FASM).

**Fig. 2:**
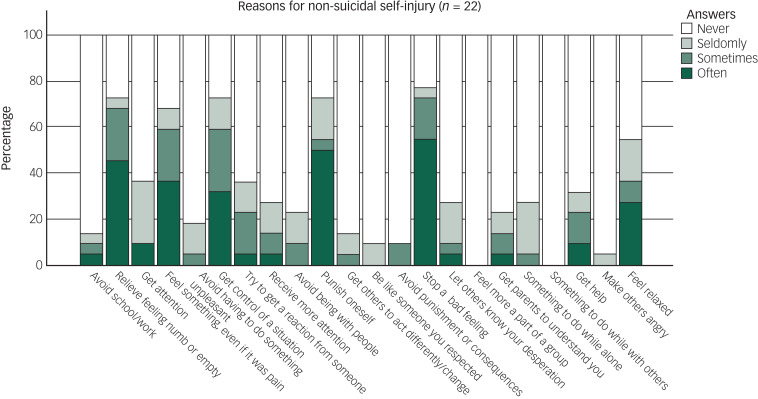
Self-reported reasons for non-suicidal self-injury, according to the Functional Assessment of Self-Mutilation (FASM).

A third of the whole sample (*n* = 20) had engaged in NSSI during the past year (15% of men and 44% of women). Almost half (44%, *n* = 28) had engaged in NSSI at some point during their lifetime (22% of men and 61% of women). The mean age at onset for NSSI was 14.5 years (s.d. 4.5, range 4–28). The most common NSSI methods were hitting oneself, picking at wounds and cutting or carving (Fig. 1). Twenty-two participants (35%) presented reasons for NSSI, the most common being to stop bad feelings, relieve feeling numb or empty, getting control of a situation or punishing oneself (Fig. 2). No participants reported ‘feeling more part of a group’ and ‘something to do while with others’ as reasons for NSSI. Among the participants with a history of NSSI, 23% experienced ‘no pain’ and 32% experienced ‘little pain’ when engaging in NSSI. ‘No time at all’ (55%) or ‘a few minutes’ (18%) were the most common time frames spent thinking about NSSI before engaging in it. There was a significant overlap between NSSI and suicidality (Table 3).

**Table 3:** Overlap of non-suicidal self-injury and history of suicidality in the total sample (*N* = 63)

	Active suicidal ideation (*n* = 54)	Suicidal plans (*n* = 40)	Attempted suicide (*n* = 16)	No suicidality^a^ (*n* = 8)
Lifetime				
NSSI (*n* = 28), *n* (%)	26 (93%)	24 (86%)	10 (36%)	1 (4%)
No NSSI (*n* = 35), *n* (%)	28 (80%)	16 (46%)	6 (17%)	7 (20%)
Past year	Active suicidal ideation (*n* = 37)	Suicidal plans (*n* = 18)	Attempted suicide (*n* = 4)	No suicidality^a^ (*n* = 24)
NSSI (*n* = 20), *n* (%)	14 (70%)	10 (50%)	2 (10%)	4 (20%)
No NSSI (*n* = 43), *n* (%)	23 (53%)	8 (19%)	2 (5%)	20 (47%)

NSSI, non-suicidal self-injury.

a. No active suicidal ideation, suicidal plans or attempted suicide during specified period. Note that categories in history of suicidality are not mutually exclusive.

A forward stepwise logistic regression was performed, with occurrence of NSSI as dependent variable and known risk factors of NSSI as covariates. The final model consisted of female sex (odds ratio 8.7, *P* < 0.01), history of suicidal plans (odds ratio 12.7, *P* < 0.01) and antisocial personality disorder (odds ratio 18.1, *P* < 0.05), and explained between 36.7% and 49.1% of the variance in the occurrence of NSSI (Cox and Snell *R^2^* = 0.367, Nagelkerke *R^2^* = 0.491). Details from the regression are available in the supplementary material.

### Alcohol and drug use

Out of the 19 participants (six men and 13 women) presenting with harmful or hazardous alcohol use and/or drug-related problems (as captured by the AUDIT/DUDIT), only six (four men and two women) had a clinical diagnosis of dependence on or harmful use of alcohol or drugs (F10 range of diagnoses in the ICD-10) according to their medical records at the follow-up.

### Possible overlap of ASD traits and depression

No differences were found in degree of ASD features/traits (ASDI score) between participants with (*n* = 31) and without (*n* = 32) an ongoing episode of depression at the time of the in-depth assessment (*t*(53.945) = 0.70, *P* = 0.49).

## Discussion

We assessed self-reported suicidal ideation, suicide attempts and NSSI in 63 adult psychiatric out-patients with ASD or subthreshold ASD diagnosed in adulthood. None of the participants had intellectual disability. Three major findings were obtained. First, the prevalence of suicidal ideation and suicide attempts were comparable to other psychiatric populations. Second, hazardous or harmful alcohol use and/or drug-related problems and history of depression were associated with history of suicide attempts. Third, prevalence and pattern (method and reasons) of NSSI were comparable to other psychiatric populations.

### Prevalence of suicidal ideation and suicide attempts

Self-reported suicidal ideation was prevalent, with 86% reporting having had thoughts of ending their own life at some point during their lifetime, 58% during the past year, 31% during the past month and 14% during the past week. Self-reported lifetime prevalence of suicide attempts was 25%. All rates were considerably higher than what has been found in the general population. In Sweden in 2020, suicidal thoughts were reported by 3% (past year) and 13% (at some point during their lifetime), whereas a history of suicide attempts (at some point during their lifetime) was reported by 5.2% of Swedish women and 3.0% of Swedish men.^51^

The prevalence rate of self-reported past-month suicidal ideation was higher in the present study (31%) than the recently reported prevalence rate of clinician-rated suicidal thoughts, threats or attempts in a Norwegian out-patient population (17.3%).^27^ The Norwegian study used data from all psychiatric patients, whereas we used data from new patients only in the present study. As suicide prevention is a highly prioritised and clearly stated treatment aim at most (if not all) psychiatric clinics, the lower rates of past-month suicidal ideation in the Norwegian study might be explained by the already established contact with psychiatric services (our participants were in the process of establishing contact with psychiatric services). The lifetime prevalence figures for suicidal ideation and suicide attempts in the present study were comparable to those recently reported in Japanese out-patients (88.6% reporting lifetime suicidal ideation and 31% reporting suicide attempts).^52^ The similarities in lifetime prevalence rates imply that adult psychiatric out-patients with autism do not differ from adult psychiatric out-patients without autism in suicidal ideation and suicide attempts from a lifetime perspective.

Compared to other adult autism populations, the rates of suicidal ideation and suicide attempts are similar to what has previously been reported in adults with autism without intellectual disability, who were diagnosed with autism in adulthood.^17,22^ Prospective studies reporting on suicidality in persons with non-intellectual disability ASD diagnosed in childhood are scarce, but those published so far point toward a substantially lower prevalence of suicidal ideation and suicide attempts when compared with those diagnosed with ASD in adulthood. For example, Gillberg et al,^6^ in a 20-year follow-up study of a cohort of men diagnosed with Asperger's syndrome in childhood (age ≤10 years), reported that only 2% had a high risk of suicidality in adult age, according to the M.I.N.I. To our knowledge, there are no other published studies reporting self-reported suicidal ideation and age of diagnosis in adults with autism without intellectual disability. When using data from patient records, incidence rates of suicide attempts have been reported to increase between 2.4 and 3.5 times in persons diagnosed with ASD after 13 years of age compared with those diagnosed earlier; figures for deaths by suicide were reported to increase even more, between 6.2 and 8.2 times.^53^

In summary, suicidal ideation and suicide attempts seem to be frequent in adults with ASD diagnosed in adulthood, at least in those referred to adult psychiatric services. No deaths by suicide were recorded in the current study group at the follow-up of medical records (a minimum of 7 months after participation). This might possibly imply that establishing a psychiatric contact is preventive of suicide in this group, although deaths by suicide are rare and larger samples are required to detect such an association.

### Factors associated with suicide attempts

Hazardous or harmful alcohol use and/or drug-related problems and number of depressive symptoms during the most severe depressive episode explained up to a third of the variance in suicide attempts. Only two out of 13 women and four out of six men presenting with harmful or hazardous alcohol use and/or drug-related problems had a clinical diagnosis of harmful use or addiction of alcohol or drugs, according to medical records. This might indicate that harmful use or addiction is not assessed thoroughly enough in adult psychiatric out-patients with autism in clinical practice, especially in women. The rate of persistent depressive disorder or the rate of having a history of depression did not differ between participants with or without a history of suicide attempts. Of the previously suggested specific risk factors for suicide attempts in adults with autism^13,18,20,21^ that could be analysed in this study (autism features/traits, depression, degree of psychiatric comorbidity and female sex), only depression was associated with history of attempted suicide.

### NSSI behaviours

NSSI was prevalent in 44% of our study sample at some point in their lifetime, and 32% had engaged in NSSI during the past year. Being female, having a history of suicide plans and occurrence of antisocial personality disorder explained up to half of the variance in occurrence of NSSI. We also found a pronounced overlap between suicidality and NSSI. The past-year prevalence of NSSI in non-clinical, general population samples varies quite substantially (14–59%) between different studies,^54–56^ which makes comparison difficult. Our results are in line with a study of a clinical psychiatric population that reported a prevalence of NSSI in about 40% of all patients during the past 6 months.^49^ Comparisons must be interpreted with caution, given that the samples differ significantly with regard to age and sex distribution. The two published studies on ASD populations so far have reported lifetime prevalence of NSSI at 50–72% and past-year prevalence of NSSI at 33–54%,^23,29^ although both studies were online surveys with self-reported ASD diagnoses. The overlap between suicidality and NSSI that we found is in line with a recent study on an adult psychiatric population^33^ and a recent study on an adult ASD population.^23^

The most common NSSI methods were hitting oneself, picking at wounds, cutting or carving oneself and biting oneself. This is in line with other studies of adults in both psychiatric and general populations,^49,56^ although the method of hitting oneself was more common in our study group. The most common self-reported underlying reasons for engaging in NSSI were to stop bad feelings, punish oneself, relieve feeling numb or empty and get control of a situation; this was similar to both psychiatric,^49^ non-psychiatric^56^ and ASD^29^ populations. Comparisons need – again – to be interpreted with caution, as the studies we compare to are from psychiatric in-patients^49^ and non-psychiatric (general population) adults who were clearly younger.^56^

Over 70% of the respondents reported that they had not spent more than a few minutes thinking about NSSI before engaging in it, indicating a very high degree of impulsiveness. This was comparable to results from non-psychiatric populations.^56^ Slightly more than half of the participants experienced no or little pain during self-injury, compared with three-fourths in non-psychiatric populations.^56^ The possibly lower proportion experiencing no pain or little pain during self-injury in our sample might be an implication of sensory hypo- or hyper-reactivity (differences in sensory reactivity is a diagnostic criterion for ASD according to the DSM-5^36^). The experience of pain has been shown to be prevalent in general during self-injury, and has been suggested to work as a negative reinforcer for self-injury behaviour.^57^ If higher rates of pain during NSSI are generalisable to all adult psychiatric out-patients with autism, it would be possible that the reinforcing mechanism in NSSI is more powerful in persons with autism.

More studies are needed to better understand the risk factors and mechanisms involved in suicidal ideation and behaviours and NSSI before we can provide targeted interventions and suicide prevention in persons with autism within psychiatric populations. Future studies need to cover larger samples to increase generalisability and enable comparisons between patients with and without autism from the same clinical population.

### Strengths and limitations

A strength of the study was that clinical assessments and diagnostic evaluations were performed by experienced clinicians. Another strength is the large catchment area. Age and sex composition of the sample are similar to the psychiatric out-patient population at the clinic where the study was performed.^9^ Limitations include a low participation rate during systematic screening (24% of the patients who screened positive for autism) and a period of unsystematic screening. This is a major limitation to generalisability; our sample could constitute a subgroup of more suicidal or less suicidal patients than that found in the studied population of adult psychiatric out-patients with autism. The absence of a comparison group is another limitation that made comparisons with other adult psychiatric out-patients difficult. The limited number of participants (<30) with prior suicide attempts lowered the statistical power, which could have resulted in false negatives in the analysis of predictive factors. Low numbers of participants with NSSI might have further decreased the representativeness of the NSSI subsample. Another limitation is that we lacked an instrument for the detection of personality syndromes other than antisocial personality syndrome. We cannot rule out that the question regarding occurrence of NSSI was difficult to interpret for the participants, because of its wording. Finally, our sample did not include any participants with intellectual disability.

In conclusion, suicidal ideation and suicide attempts were common in this sample of adult psychiatric out-patients with ASD diagnosed in adulthood. Factors associated with suicide attempts were high self-reported consumption of alcohol and/or drugs and number of depressive symptoms during the most severe depressive episode. NSSI was prevalent, and methods of and reasons for engaging in NSSI were reported to be similar to those reported in other psychiatric populations. The findings suggest that assessment of alcohol and drug consumption and depression is important to detect suicidal ideation, and that alcohol and drug consumption might be especially overlooked in women. Underlying mechanisms of NSSI in psychiatric patients with autism seem similar to other psychiatric populations, and clinicians should conceptualise treatment of NSSI similarly, regardless of whether the patient has autism.

## Supporting information

Nyrenius et al. supplementary material Nyrenius et al. supplementary material

## Data Availability

The data that support the findings of this study are available from the corresponding author, J.N., upon reasonable request. The data are not publicly available due to their containing information that could compromise the privacy of research participants.
