# Supplementary material for: Autism in adult psychiatric out-patients: self-reported suicidal ideation, suicide attempts and non-suicidal self-injury
Source: BJPsych Open. 2023 Sep 7;9(5):e167. doi: 10.1192/bjo.2023.553 (PMC10594204; doi:10.1192/bjo.2023.553)
Supplement: Nyrenius et al. supplementary material [file S2056472423005537sup001.docx]

**Supplementary material**

**Table S1**. Forward stepwise logistic regression between suicide attempts and known risk factors

| Covariate | Odds Ratio | *P* value |
| --- | --- | --- |
| Variables not in the equation |  |  |
| Age |  | 0.35 |
| Sex |  | 0.58 |
| Previous dependence on income support |  | 0.90 |
| ASDI score |  | 0.14 |
| Number of non-mood disorders^1^ |  | 0.23 |
| IQ estimate |  | 0.95 |
| PSI |  | 0.92 |
| Having close friends^2^ |  | 0.33 |
| Variables in the equation |  |  |
| Hazardous or harmful alcohol use, and/or drug-related problems^3^ | 6.0 | <0.01 |
| Number of depression symptoms from most severe episode | 2.2 | <0.05 |

IQ = Intelligence Quotient; PSI = Processing Speed Index; ^1^ According to M.I.N.I and self-reported / clinically assessed tic disorder; ^2^ Self-reported “do you have close friends – persons you spend time with on a regular basis and feel that you can trust?”; ^3^ Score ≥ 8 (males) or ≥ 6 (females) on AUDIT (Alcohol Use Disorders Identification Test) and/or score ≥ 6 (males) or ≥ 2 (females) on DUDIT (Drug Use Disorder Identification Test).

**Table S2**. Forward stepwise logistic regression between non-suicidal self-injury and known risk factors

| Covariate | Odds Ratio | *P* value |
| --- | --- | --- |
| Variables not in the equation |  |  |
| Age |  | 0.09 |
| History of active suicidal ideation |  | 0.78 |
| History of suicide attempt(s) |  | 0.66 |
| ASDI result |  | 0.30 |
| Occurrence of ADHD (any subtype)^1^ |  | 0.67 |
| Variables in the equation |  |  |
| Female sex | 8.7 | <0.01 |
| History of suicidal plans | 12.7 | <0.01 |
| Antisocial personality disorder^2^ | 18.1 | <0.05 |

ASDI = Asperger Syndrome (and high-functioning autism) Diagnostic Interview; ^1^According to M.I.N.I section Q; ^2^According to M.I.N.I section P.

**Table S3**. Multi-collinearity analysis: Spearman correlations between variables used in stepwise logistic regression with occurrence of suicide attempts as dependent variable

|  | A | B | C | D | E | F | G | H | I | J |
| --- | --- | --- | --- | --- | --- | --- | --- | --- | --- | --- |
| A | 1 | -0.27* | 0.34* | -0.24 | 0.17 | -0.02 | -0.10 | -0.31* | 0.07 | 0.12 |
| B | -0.27* | 1 | -0.15 | -0.01 | 0.19 | 0.07 | 0.17 | 0.03 | 0.15 | 0.22 |
| C | 0.34* | -0.15 | 1 | 0.13 | 0.14 | 0.26* | -0.22 | -0.18 | 0.30* | 0.35* |
| D | -0.24 | -0.01 | 0.13 | 1 | -0.09 | 0.02 | -0.12 | -0.05 | <0.01 | -0.13 |
| E | 0.17 | 0.19 | 0.14 | -0.09 | 1 | -0.17 | -0.15 | 0.02 | 0.09 | 0.37** |
| F | -0.02 | 0.07 | 0.26* | 0.02 | -0.17 | 1 | 0.79** | -0.15 | -0.20 | 0.02 |
| G | -0.10 | 0.17 | -0.22 | -0.12 | -0.15 | 0.79** | 1 | -0.15 | -0.19 | 0.02 |
| H | -0.31* | 0.03 | -0.18 | -0.05 | 0.02 | -0.15 | -0.15 | 1 | 0.08 | -0.06 |
| I | 0.07 | 0.15 | 0.30* | <0.01 | 0.09 | -0.20 | -0.19 | 0.08 | 1 | 0.18 |
| J | 0.12 | 0.22 | 0.35* | -0.13 | 0.37** | 0.02 | 0.02 | -0.06 | 0.18 | 1 |

*: Statistically significant on .05 level; **: Statistically significant on .01 level; A: Age; B: Sex; C: Previous dependence on income support; D: ASDI (Asperger Syndrome (and high-functioning autism) Diagnostic Interview) score; E: Number of current non-mood disorders; F: IQ (Intelligence Quotient) estimate; G: PSI (Processing Speed Index); H: Having close friends; I: Hazardous or harmful alcohol use, and/or drug-related problems; J: Number of depression symptoms from most severe episode

**Table S4**. Multi-collinearity analysis: Spearman correlations between variables used in stepwise logistic regression with occurrence of non-suicidal self-injury as dependent variable

|  | A | B | C | D | E | F | G | H |
| --- | --- | --- | --- | --- | --- | --- | --- | --- |
| A | 1 | -0.27* | <0.01 | 0.13 | 0.09 | -0.24 | 0.05 | 0.03 |
| B | -0.27* | 1 | -0.08 | 0.01 | 0.14 | -0.01 | -0.07 | 0.10 |
| C | <0.01 | -0.08 | 1 | 0.44** | 0.24 | -0.18 | -0.19 | <0.01 |
| D | 0.13 | 0.01 | 0.44** | 1 | 0.44* | -0.13 | 0.02 | 0.06 |
| E | 0.09 | 0.14 | 0.24 | 0.44* | 1 | -0.20 | 0.03 | 0.14 |
| F | -0.24 | -0.01 | -0.18 | -0.13 | -0.20 | 1 | 0.09 | -0.27* |
| G | 0.05 | -0.07 | -0.19 | 0.02 | 0.03 | 0.09 | 1 | 0.14 |
| H | 0.03 | 0.10 | <0.01 | 0.06 | 0.14 | -0.27* | 0.14 | 1 |

*: Statistically significant on .05 level; **: Statistically significant on .01 level; A: Age; B: Sex; C: History of active suicidal ideation; D: History of suicidal plans; E: History of suicide attempt; F: ASDI (Asperger Syndrome (and high-functioning autism) Diagnostic Interview) score; G: Occurrence of ADHD (Any subtype); H: Antisocial personality disorder.
